# Tumor necrosis factor inhibitors enhance corticosteroid therapy for Stevens-Johnson syndrome and toxic epidermal necrolysis linked to immune checkpoint inhibitors: a prospective study

**DOI:** 10.3389/fimmu.2024.1421684

**Published:** 2024-08-07

**Authors:** Chun-Xia He, Lan Guo, Tao Qu, Hong-Zhong Jin

**Affiliations:** Department of Dermatology, State Key Laboratory of Complex Severe and Rare Diseases, Peking Union Medical College Hospital, Chinese Academy of Medical Sciences and Peking Union Medical College, National Clinical Research Center for Dermatologic and Immunologic Diseases, Beijing, China

**Keywords:** immune checkpoint inhibitors, immune-related adverse events, severe cutaneous adverse reactions, Stevens-Johnson Syndrome, toxic epidermal necrolysis, tumor necrosis factor inhibitors

## Abstract

**Introduction:**

Immune-related epidermal necrolysis (irEN), including Stevens-Johnson Syndrome (SJS) and toxic epidermal necrolysis (TEN), represents a potentially lethal reaction to immune checkpoint inhibitors. An optimal treatment strategy remains undefined. This study evaluates the effectiveness and safety of combination therapy with corticosteroids and tumor necrosis factor inhibitors (TNFi) in treating irEN patients.

**Methods:**

In this single-center, prospective, observational study, patients with irEN received either corticosteroid monotherapy or a combination therapy of corticosteroids and TNFi (etanercept for SJS, infliximab for TEN). The primary endpoint was re-epithelization time, with secondary endpoints including corticosteroid exposure, major adverse event incidence, acute mortality rates, and biomarkers indicating disease activity and prognosis. The study was registered at the Chinese Clinical Trial Registry (ChiCTR2100051052).

**Results:**

Thirty-two patients were enrolled (21 SJS, 11 TEN); 14 received combination therapy and 18 received corticosteroid monotherapy. IrEN typically occurred after 1 cycle of ICI administration, with a median latency of 16 days. Despite higher SCORTEN scores in the combination group (3 vs. 2, p = 0.008), these patients experienced faster re-epithelization (14 vs. 21 days; p < 0.001), shorter corticosteroid treatment duration (22 vs. 32 days; p = 0.005), and lower prednisone cumulative dose (1177 mg vs. 1594 mg; p = 0.073). Major adverse event rates were similar between groups. Three deaths occurred due to lung infection or disseminated intravascular coagulation, with mortality rates for both groups lower than predicted. Potential risk factors for increased mortality included continuous reduction in lymphocyte subset counts (CD4^+^ T cells, CD8^+^ T cells, natural killer cells) and consistent rises in inflammatory markers (serum ferritin, interleukin-6, TNF-α). Re-epithelization time negatively correlated with body mass index and positively correlated with epidermal detachment area and serum levels of interleukin-6 and TNF-α.

**Conclusions:**

Corticosteroids combined with TNFi markedly promote re-epithelization, reduce corticosteroid use, and decrease acute mortality in irEN patients without increasing major adverse events, offering a superior alternative to corticosteroid monotherapy. Inflammatory markers and lymphocyte subsets are valuable for assessing disease activity and prognosis.

## 1 Introduction

The advent of immune checkpoint inhibitors (ICIs) has revolutionized cancer therapy, offering substantial benefits across various tumor types. However, these agents are also associated with a spectrum of immune-related adverse events (irAEs), of which cutaneous manifestations are the most prevalent (1–4). Among these, immune-related epidermal necrolysis (irEN) stands out as particularly severe, characterized by widespread erythemas and blisters, accompanied by high fever and mucositis, with mortality rates reaching up to 40% (5, 6). This spectrum disorder is classified into three types based on the extent of epidermal detachment: Stevens-Johnson syndrome (SJS), which involves less than 10% of the body surface area (BSA); toxic epidermal necrolysis (TEN), which affects more than 30% of BSA; and SJS/TEN overlap syndrome (10%-30% of BSA involved) (7). The management of irEN presents considerable challenges due to its rarity and potentially fatal nature, necessitating a multidisciplinary approach to treatment.

Currently, the primary management strategy for irEN involves corticosteroid therapy, supplemented by interventions such as intravenous immunoglobulins (IVIG), cyclosporine, and tumor necrosis factor inhibitors (TNFi) based on expert consensus rather than robust evidence (8, 9). However, recent studies suggest potential efficacy and safety of TNFi in treating irEN. Preclinical studies have indicated that TNFi may mitigate immune-related colitis while enhancing anti-tumor effect of ICIs (10, 11). Clinical experiences from case reports and retrospective studies further support the use of TNFi in managing immune-related colitis and pneumonitis (12, 13). Furthermore, evidence from a randomized controlled trial and systematic reviews underscores the safety and benefits of TNFi, either as monotherapy or combined with corticosteroids, in treating epidermal necrolysis induced by conventional drugs (14–16). Nonetheless, specific research on the role of TNFi in irEN remains limited.

Our study aims to evaluate the effectiveness and safety of the combination therapy with corticosteroids and TNFi for the treatment of irEN. Specifically, we seek to determine if this approach accelerates skin re-epithelization, reduces corticosteroid exposure, and decreases acute mortality rates, without increasing major treatment-related adverse events compared to corticosteroid monotherapy. We also explored the biomarkers associated with disease activity and prognosis.

## 2 Methods

### 2.1 Study design and participants

This prospective, observational study was conducted at Peking Union Medical College Hospital, Beijing, from September 2021 to March 2024. Adult patients diagnosed with irEN were enrolled. Based on the severity of their condition and contraindications to TNFi, patients were allocated to receive either corticosteroid monotherapy or a combination of corticosteroids and TNFi. Those diagnosed with TEN also received IVIG. The study's primary endpoint was re-epithelization time, with secondary endpoints including the corticosteroid exposure, acute mortality rates, incidence of major treatment-related adverse events, and biomarkers indicative of disease activity and prognosis.

Eligible participants met the following criteria: (1) aged between 18 and 75 years; (2) diagnosed with epidermal necrolysis according to the Bastuji-Garin et al. classification (7); (3) developed epidermal necrolysis after ICI administration, considered by the investigators as possibly, probably, or definitely linked to ICIs; (4) in the acute phase, characterized by new erythemas, blisters, or expanding epidermal detachment; (5) tested negative for serum anti-desmoglein and anti-BP180 antibodies.

### 2.2 Procedures and outcomes

Baseline assessments included:

1. Hematological tests: complete blood count, blood chemistry.

2. Inflammatory markers: C-reactive protein (CRP), serum ferritin (SF), interleukin-6 (IL-6), tumor necrosis factor-α (TNF-α).

3. Peripheral blood lymphocyte subsets: CD3^+^, CD4^+^, CD8^+^ T cells, CD56^+^ CD16^+^ natural killer (NK) cells.

4. Skin biopsies when necessary.

All patients received 1-2 mg/kg/day of methylprednisolone within 24 hours of admission, along with supportive care including wound management, fluid resuscitation, nutritional support, and infection prevention and control. TNFi were considered for patients with a severity-of-illness score for toxic epidermal necrolysis (SCORTEN) (17) ≥ 3, epidermal detachment ≥ 10% of BSA, or worsening condition despite 2-3 days of corticosteroid therapy, provided there was no heart failure, active hepatitis, or severe infection present. For SJS, etanercept was administered subcutaneously at 50 mg twice weekly until disease stabilization, whereas TEN cases received a single intravenous dose of infliximab at 5 mg/kg plus IVIG (0.4-0.6 g/kg/day, total ≥ 2 g/kg).

Etanercept was chosen for SJS based on randomized controlled trial evidence demonstrating its efficacy and safety in epidermal necrolysis (16). Infliximab was preferred for TEN due to its stronger and faster TNF-α inhibition, suitable for TEN's rapid progression. Notably, methylprednisolone pulse therapy was not employed in our cohort. Treatment choices were based on our center's clinical experience and the study's observational nature.

Corticosteroid tapering was initiated upon disease stabilization, indicated by no new erythemas or blisters and the start of re-epithelization. The recovery phase assessments, typically conducted 1-2 weeks after stabilization, included complete blood counts, biochemical analysis, and re-evaluation of inflammatory markers and lymphocyte subsets. All participants were followed up for at least 8 weeks.

The primary endpoint of the study was re-epithelization time, measured from the start of corticosteroid treatment to the complete healing of all erosions. Secondary endpoints included: (1) the corticosteroid duration and cumulative dose; (2) acute mortality rates, including both predicted and observed mortalities. Observed mortality rate was the proportion of patients who died within 8 weeks. Predicted mortality rate was calculated as e^logit^ / (1 + e^logit^), where logit = -4.448 + 1.237 × SCORTEN (17); (3) the incidence of major treatment-related adverse events, such as infections, gastrointestinal bleeding, and organ failure; (4) biomarkers indicative of disease activity and prognosis.

The study was approved by the Ethics Committee of Peking Union Medical College Hospital (ZS-3094, I-23PJ020, I-23PJ558, I-23PJ2115) and registered at the Chinese Clinical Trial Registry in September 2021 (ChiCTR2100051052). All participants provided written informed consent.

### 2.3 Statistical analysis

For normally distributed continuous variables, the mean ± standard deviation is presented, and differences between groups are analyzed using a two-tailed t-test. Non-normally distributed continuous variables are summarized using the median (25th and 75th percentiles), with group differences assessed using the Mann-Whitney U test. Categorical variables are expressed as counts (percentages), and intergroup differences are analyzed with Fisher's exact test or Fisher-Freeman-Halton exact test. Re-epithelization time was plotted using Kaplan-Meier curve and Log-rank test was used to compare the differences between groups. The paired sample t-test or Wilcoxon signed-rank test is used for changes in laboratory parameters from acute to recovery phase. Pearson's correlation coefficient or Spearman's rank correlation coefficient was used to assess the relationship between two variables. Specific statistical methods can be found in the corresponding figures and tables. All statistical analyses and graphs were carried out by SPSS 27.0 and GraphPad Prism 9, and p < 0.05 was considered significant.

## 3 Results

### 3.1 Patient disposition and baseline characteristics

From September 2021 to December 2023, our study enrolled 32 patients diagnosed with irEN. Of these, 21 (66%) presented with SJS and 11 (34%) with TEN. Patients were allocated into two treatment arms: a combination group (n = 14) receiving corticosteroids plus TNFi, and a corticosteroid-only group (n = 18). Within the combination group, etanercept was administered to 9 SJS patients, while infliximab was given to 5 TEN patients. All participants completed the study, with 29 survivors and 3 fatalities recorded. Comprehensive data on baseline characteristics, clinical manifestations, treatment protocols, and patient outcomes are presented in **Supplementary Tables 1**-**3**.

Of these, 18 (56%) were male, with an average age of 59 years and a mean body mass index (BMI) of 23.1 kg/m². The most common malignancies were lung cancer, gastrointestinal cancer, and hepatocellular carcinoma. The frequently used ICIs included sintilimab, pembrolizumab, camrelizumab, and tislelizumab. IrEN typically occurred after 1 cycle of ICI treatment, with a median latency of 16 days. All patients had received concurrent medications or therapy within 4 weeks prior onset, with chemotherapy and targeted agents being the predominant. Clinically, 26 patients (81%) exhibited fever, with a peak temperature of 39.0°C. Mucositis was present in 27 patients (84%), most commonly affecting the oral cavity and eyes. Generalized erythemas were observed in all patients, with a median epidermal detachment covering 8% of BSA. The median SCORTEN was 3, predicting a mortality rate of 32%. Regarding treatment, the median prednisone equivalent dose was 1.5 mg/kg/day, and the median cumulative dose of IVIG was 3.1 g/kg. No significant differences were found between the groups in demographics, clinical manifestations, laboratory findings, or treatment details. However, SCORTEN and predicted mortality rate were significantly higher in the combination group (3 vs. 2, p = 0.008, and 32% vs. 12%, p = 0.008, respectively). Detailed information is presented in **Table 1** and **Supplementary Table 4**.

**Table 1:** Comparison of baseline characteristics and treatment regimens between combination and corticosteroid groups in patients with immune-related epidermal necrolysis.

	Total cohort (n = 32)	Combination group (n = 14)	Corticosteroid group (n = 18)	p value
Age, years	59 ± 11	62 ± 6	56 ± 14	0.175
Sex, male	18 (56%)	8 (57%)	10 (56%)	0.928
Body mass index, kg/m^2^	23.1 ± 4.0	23.0 ± 3.5	23.2 ± 4.5	0.911
Malignancies				
Lung cancer	13 (41%)	7 (50%)	6 (33%)	0.521
Gastrointestinal cancer	9 (28%)	4 (29%)	5 (28%)	
Hepatocellular carcinoma	6 (19%)	1 (7%)	5 (28%)	
Other malignancies^a^	4 (12%)	2 (14%)	2 (11%)	
Culprit ICIs				
Sintilimab	13 (41%)	5 (36%)	8 (44%)	0.252
Pembrolizumab	7 (22%)	5 (36%)	2 (11%)	
Camrelizumab	5 (16%)	2 (14%)	3 (17%)	
Tislelizumab	3 (9%)	0	3 (17%)	
Other ICIs^b^	4 (12%)	2 (14%)	2 (11%)	
Cycles from ICI start to onset	1 (1, 3)	1 (1, 2)	1 (1, 3)	0.699
Latency from ICI start to onset, days	16 (8, 54)	13 (8, 52)	19 (9, 108)	0.482
Concurrent treatments within 4 weeks prior onset				
Chemotherapy ± targeted drugs	17 (53%)	10 (71%)	7 (39%)	0.260
Targeted drugs	9 (28%)	2 (14%)	7 (39%)	
Radiation therapy	2 (6%)	1 (7%)	1 (6%)	
Antibiotics	2 (6%)	1 (7%)	1 (6%)	
Other medications^c^	2 (6%)	0	2 (11%)	
Clinical manifestations				
Stevens-Johnson syndrome	21 (66%)	9 (64%)	12 (67%)	0.888
Toxic epidermal necrolysis	11 (34%)	5 (36%)	6 (33%)	
Patients with fever	26 (81%)	12 (86%)	14 (78%)	0.672
Peak body temperature, °C	39.0 ± 0.6	38.9 ± 0.7	39.1 ± 0.6	0.375
Patients with mucositis	27 (84%)	12 (86%)	15 (83%)	1.000
Erythemas, % of BSA	80 ± 8	78 ± 8	82 ± 8	0.161
Epidermal detachment, % of BSA	8 (5, 35)	9 (5, 36)	5 (4, 35)	0.488
SCORTEN (range 0-7)	3 (2, 4)	3 (3, 4)	2 (2, 3)	0.008
Predicted mortality, %	32 (12, 55)	32 (32, 68)	12 (12, 32)	0.008
Treatment regimens				
Prednisone equivalent dose, mg/day	100 (100, 100)	100 (94, 100)	100 (100, 100)	0.861
Prednisone equivalent dose, mg/kg/day	1.5 (1.3, 1.8)	1.5 (1.4, 1.8)	1.5 (1.3, 1.8)	0.909
Combined with IVIG	11 (34%)	5 (36%)	6 (33%)	1.000
IVIG cumulative dose, g/kg	3.1 (2.9, 5.5)	3.1 (2.9, 6.4)	3.4 (2.6, 5.8)	0.931

Data are presented as mean ± standard deviation, median (25th and 75th percentiles), or counts (percentages) as appropriate. P values were calculated using two-tailed t-test for normally distributed continuous variables, Mann-Whitney U test for non-normally distributed continuous variables, and Fisher's exact test or Fisher-Freeman-Halton exact test for categorical variables. ^a^Other malignancy types include one case each of thymoma, retroperitoneal carcinoma, scrotum adenocarcinoma, and malignant mesothelioma. ^b^Other ICIs include one case each of serplulimab, toripalimab, durvalumab and ipilimumab. ^c^Other medications include one case each of Chinese herb and nivolumab. BSA, body surface area; ICIs, immune checkpoint inhibitors; IVIG, intravenous immunoglobulin; SCORTEN, severity-of-illness score for toxic epidermal necrolysis.

Compared to SJS patients, those with TEN exhibited significantly lower BMI (20.2 vs. 24.6 kg/m^2^, p = 0.002), more extensive epidermal detachment (40% vs. 5% of BSA, p < 0.001), higher SCORTEN (4 vs. 2, p < 0.001), and lower serum albumin levels (33 vs. 39 g/L, p < 0.001), indicating greater disease severity and worse general condition. Furthermore, TEN patients showed elevated levels of inflammatory markers and decreased lymphocyte subset counts, as detailed in **Supplementary Table 5**.

### 3.2 Primary endpoint: re-epithelization time in healed patients

**Figure 1:**
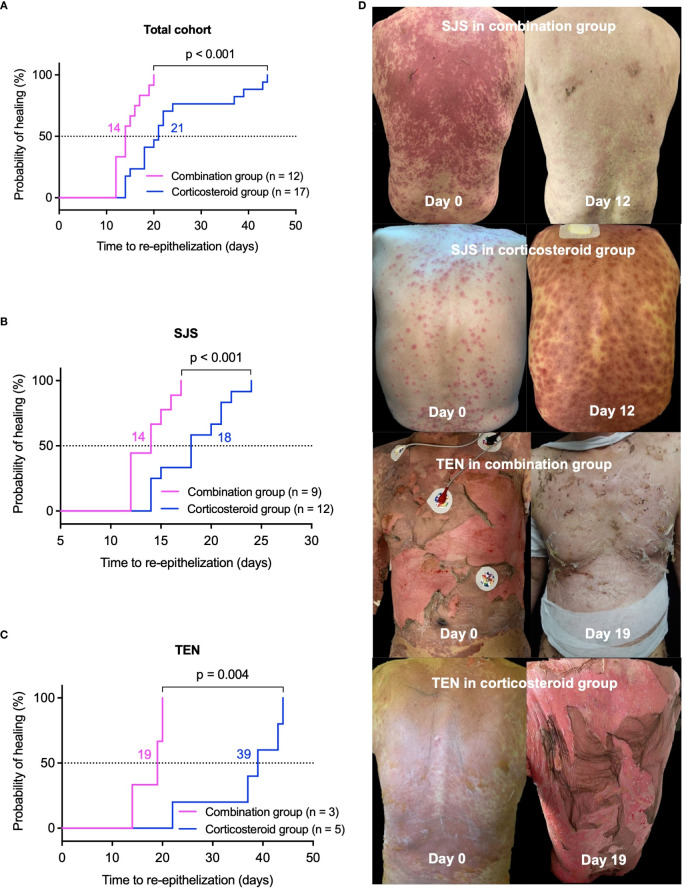
Comparison of re-epithelization time between combination and corticosteroid groups in healed patients. Kaplan-Meier survival curves illustrate the probability of re-epithelization over time. **(A)** Total cohort, **(B)** SJS patients, and **(C)** TEN patients. Panel **(D)** features representative photographs of lesions before and after treatment in patients with SJS or TEN from the combination and corticosteroid groups. P values were determined by Log-rank test in A-C. SJS, Stevens-Johnson syndrome; TEN, toxic epidermal necrolysis.

In the healed cohort of 29 patients, median re-epithelization time was significantly shorter in the combination group, 14 (12, 17) days versus 21 (17, 31) days in the corticosteroid group (p < 0.001, **Figure 1A**). For SJS patients, the combination group experienced a median re-epithelization time of 14 (12, 16) days, compared to 18 (14, 21) days in the corticosteroid group (p < 0.001, **Figure 1B**). TEN patients in the combination group showed a median re-epithelization time of 19 (17, 20) days, significantly shorter than 39 (30, 44) days in the corticosteroid group (p = 0.004, **Figure 1C**). **Figure 1D** displays representative photographs of skin lesions before and after treatment in SJS and TEN patients from both groups.

### 3.3 Secondary endpoints

#### 3.3.1 Corticosteroid duration and cumulative dosage in healed patients

**Figure 2:**
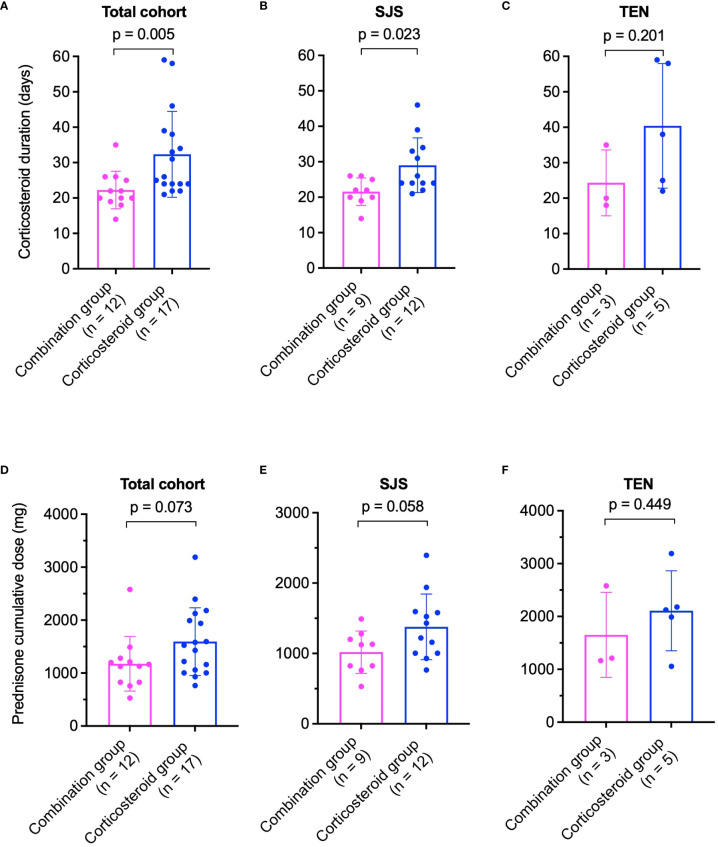
Comparison of corticosteroid treatment durations **(A–C)** and cumulative prednisone doses **(D–F)** between combination and corticosteroid groups in healed patients. **(A)** Total cohort, **(B)** SJS patients, and **(C)** TEN patients for treatment duration. **(D)** Total cohort, **(E)** SJS patients, and **(F)** TEN patients for cumulative prednisone dose. P values were determined by Mann-Whitney U test in **(A, B)**, and two-tailed t-test in **(C–F)**. SJS, Stevens-Johnson syndrome; TEN, toxic epidermal necrolysis.

In the healed cohort, average corticosteroid duration was significantly shorter in the combination group (22 ± 5 days) compared to the corticosteroid group (32 ± 12 days, p = 0.005, **Figure 2A**). For SJS patients, corticosteroid duration in the combination group was 22 ± 4 days, significantly less than 29 ± 8 days in the corticosteroid group (p = 0.023, **Figure 2B**). Among TEN patients, treatment lasted 24 ± 9 days in the combination group versus 40 ± 18 days in the corticosteroid group (p = 0.201, **Figure 2C**).

The cumulative prednisone equivalent dose was lower in the combination group across the healed cohort (1177 ± 515 mg vs. 1594 ± 640 mg, p = 0.073, **Figure 2D**). In SJS patients, cumulative doses were 1018 ± 302 mg in the combination group and 1379 ± 466 mg in the corticosteroid group (p = 0.058, **Figure 2E**). For TEN patients, doses were 1651 ± 804 mg in the combination group and 2109 ± 757 mg in the corticosteroid group (p = 0.449, **Figure 2F**).

#### 3.3.2 Acute mortality rates

In the entire cohort of 32 patients, the predicted mortality rate was 32% (12%, 55%), while the observed rate was 9% (3/32). The combination group had a predicted mortality of 32% (32%, 68%) and an observed rate of 14% (2/14). The corticosteroid group's predicted mortality was 12% (12%, 32%) with an observed rate of 6% (1/18).

Among SJS patients, predicted mortality rates were 32% (22%, 32%) for the combination group and 12% (12%, 12%) for the corticosteroid group, with no observed deaths in either group. In TEN patients, the combination group's predicted mortality rate was 85% (62%, 90%), with an observed rate of 40% (2/5), while the corticosteroid group had a predicted mortality rate of 32% (32%, 62%), with an observed rate of 17% (1/6).

**Figure 3:**
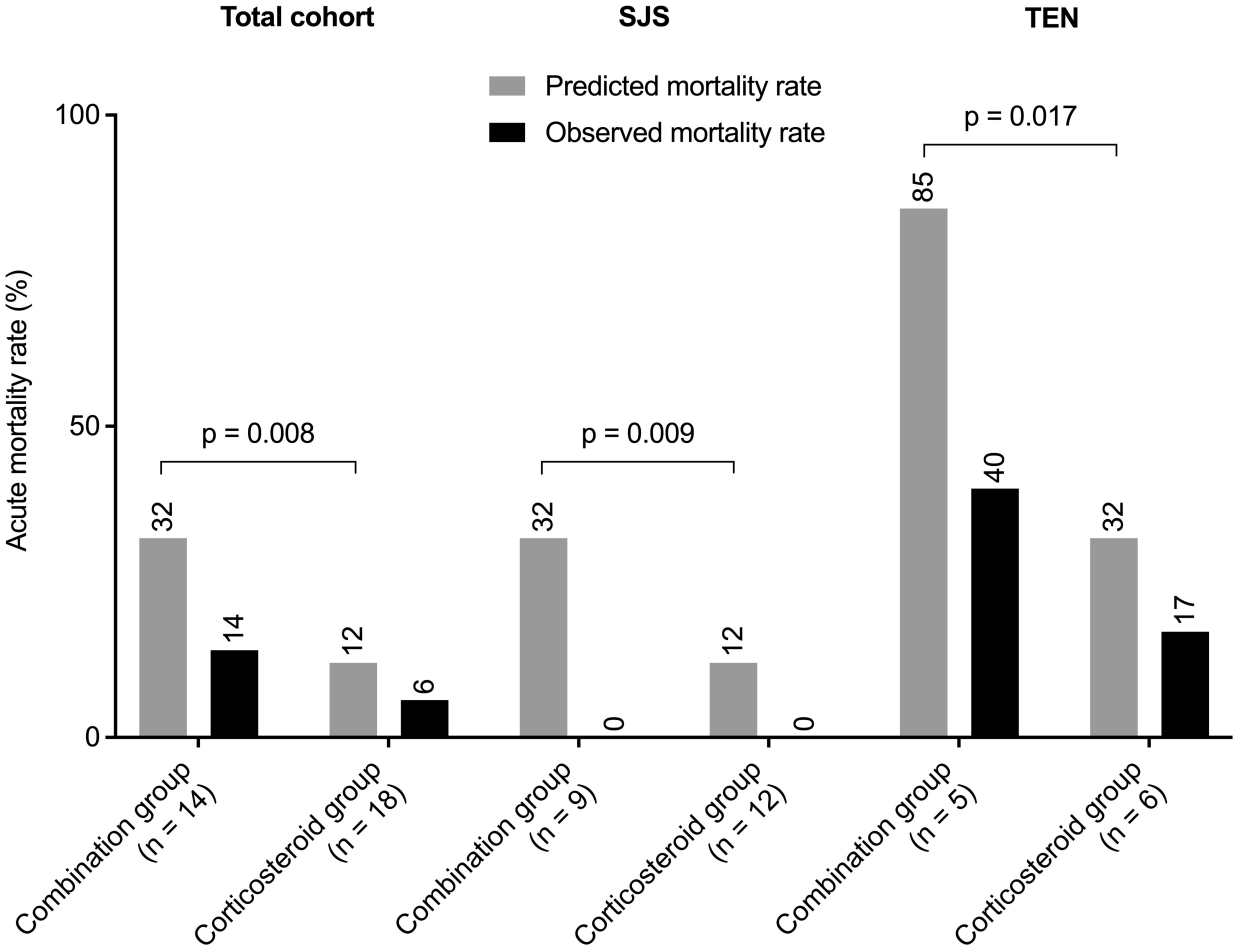
Predicted versus observed mortality rates for the entire cohort, and separately for patients with SJS and TEN. P values were determined by Mann-Whitney U test. SJS, Stevens-Johnson syndrome; TEN, toxic epidermal necrolysis.

Predicted mortality rates were significantly higher in the combination group across all cohorts. However, differences in observed mortality rates between groups were not statistically significant. Detailed data are presented in **Figure 3**. Causes of death were pulmonary infection in 2 cases (due to *Aspergillus* species and pan-resistant *Acinetobacter baumannii*, respectively) and disseminated intravascular coagulation in 1 case.

#### 3.3.3 Major treatment-related adverse events

Major treatment-related adverse events included infections, disseminated intravascular coagulation, gastrointestinal bleeding, and respiratory failure. The incidence of these events did not differ significantly between groups. However, infections occurred earlier in the combination group (8 vs. 21 days, p = 0.114). Detailed data are presented in **Table 2**.

**Table 2:** Major treatment-related adverse events occurred in patients with immune-related epidermal necrolysis.

	Total cohort (n = 32)	Combination group (n = 14)	Corticosteroid group (n = 18)	p value
Infections	7 (22%)	3 (21%)	4 (22%)	1.000
Lung infection	5 (16%)	2 (14%)	3 (17%)	1.000
Sepsis	3 (9%)	2 (14%)	1 (6%)	0.568
Disseminated intravascular coagulation	3 (9%)	2 (14%)	1 (6%)	0.568
Gastrointestinal bleeding	2 (6%)	1 (7%)	1 (6%)	1.000
Respiratory failure	2 (6%)	1 (7%)	1 (6%)	1.000
Time from corticosteroid start to infection, days	20 (8, 21)	8 (7, 14)	21 (14, 29)	0.114
Time from corticosteroid start to death, days	20 (18, 23)	18 (15, 20)	26 (n = 1)	1.000

Data are presented as median (25th and 75th percentiles) or counts (percentages) as appropriate. P values were calculated using Mann-Whitney U test for non-normally distributed continuous variables, and Fisher's exact test for categorical variables.

#### 3.3.4 Biomarkers indicative of disease activity and prognosis

##### 3.3.4.1 Factors associated with re-epithelization time in healed patients

**Figure 4:**
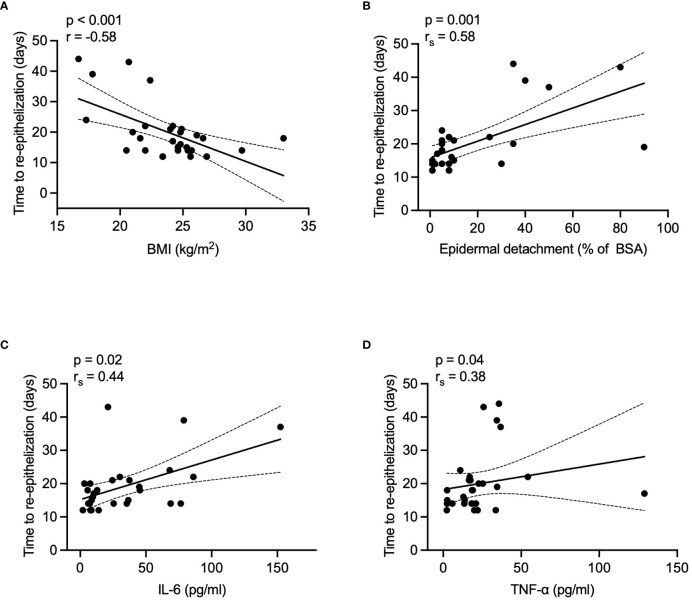
Correlations between re-epithelization time and various factors in healed patients. **(A)** BMI, **(B)** epidermal detachment area, **(C)** serum IL-6 levels, and **(D)** TNF-α levels. P values were determined by Pearson's correlation coefficient in A, or Spearman's rank correlation coefficient in B-D. BMI, body mass index; BSA, body surface area; IL-6, interleukin-6; TNF-α, tumor necrosis factor-α.

Re-epithelization time negatively correlated with BMI (r = -0.58, p < 0.001) and positively correlated with epidermal detachment area (r_s_ = 0.58, p = 0.001), serum IL-6 (r_s_ = 0.44, p = 0.02), and TNF-α (r_s_ = 0.38, p = 0.04) levels during the acute phase. Details are presented in **Figure 4**. No significant correlations were found with patient demographics, SCORTEN, or other laboratory parameters (data not shown).

##### 3.3.4.2 Biomarkers reflecting disease activity in healed patients

**Figure 5:**
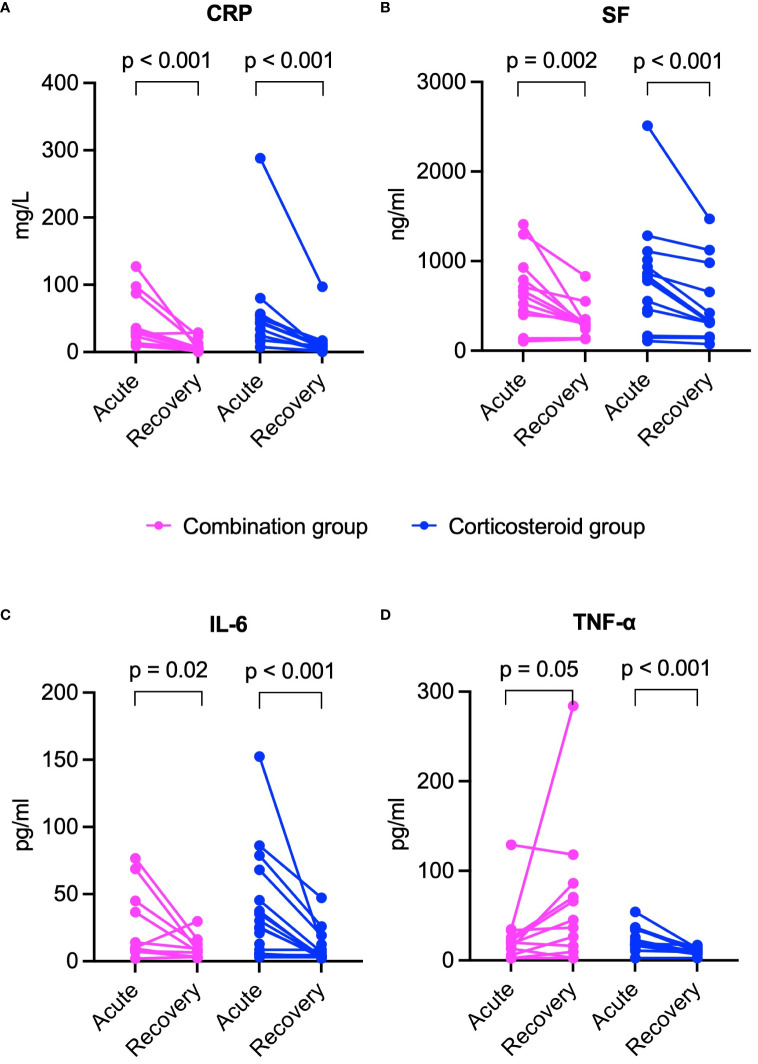
Comparison of inflammatory marker changes from acute to recovery phase between combination and corticosteroid groups in healed patients. **(A)** CRP, **(B)** SF, **(C)** IL-6, and **(D)** TNF-α. P values were determined by Wilcoxon signed-rank test. CRP, C-reactive protein; IL-6, interleukin-6; SF, serum ferritin; TNF-α, tumor necrosis factor-α.

**Figure 6:**
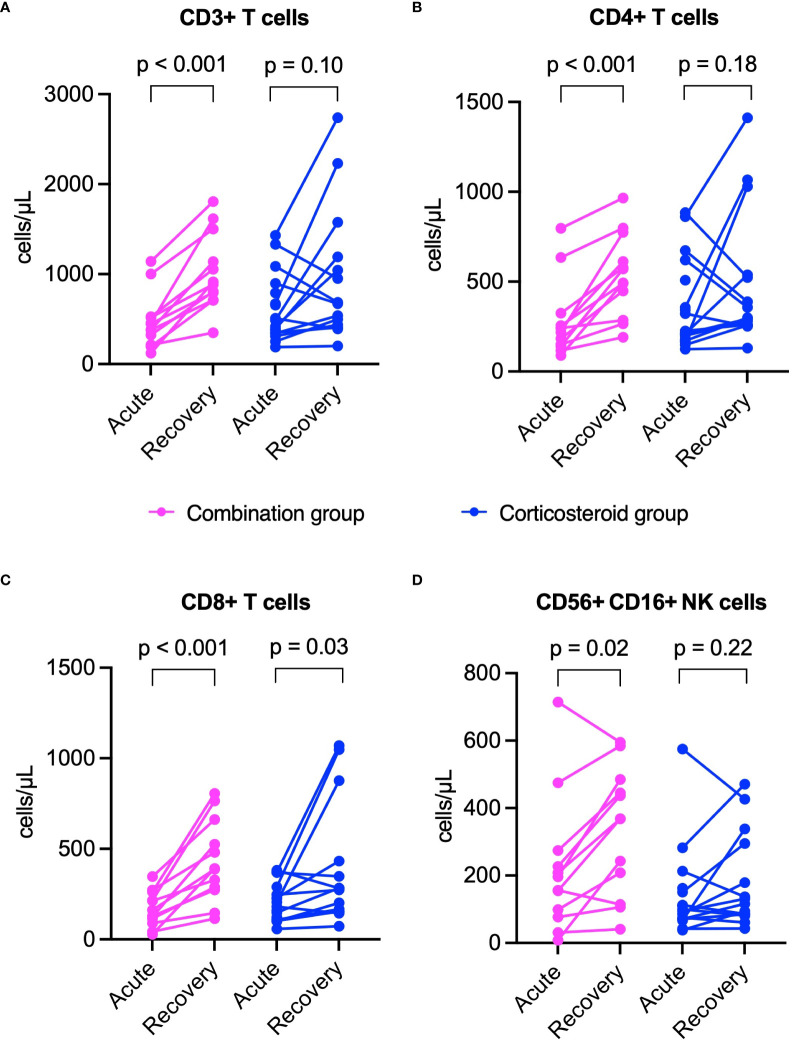
Comparison of lymphocyte subset count changes from acute to recovery phase between combination and corticosteroid groups in healed patients. **(A)** CD3^+^ T cells, **(B)** CD4^+^ T cells, **(C)** CD8^+^ T cells, and **(D)** NK cells. P values were determined by Wilcoxon signed-rank test in A and D, or paired sample t-test in B and **(C)** NK, natural killer.

In both groups, inflammatory markers including CRP, SF, and IL-6 significantly declined from acute to recovery phase (p < 0.05 for all, **Figures 5A-C**). However, TNF-α levels decreased significantly only in the corticosteroid group, while showing an upward trend in the combination group (**Figure 5D**). The combination group showed significant increases in lymphocyte subset counts (CD3^+^, CD4^+^, CD8^+^ T cells, NK cells) during recovery. The corticosteroid group showed similar trends, but only CD8^+^ T cell increase was statistically significant (**Figures 6A-D**).

##### 3.3.4.3 Potential risk factors for mortality

**Figure 7:**
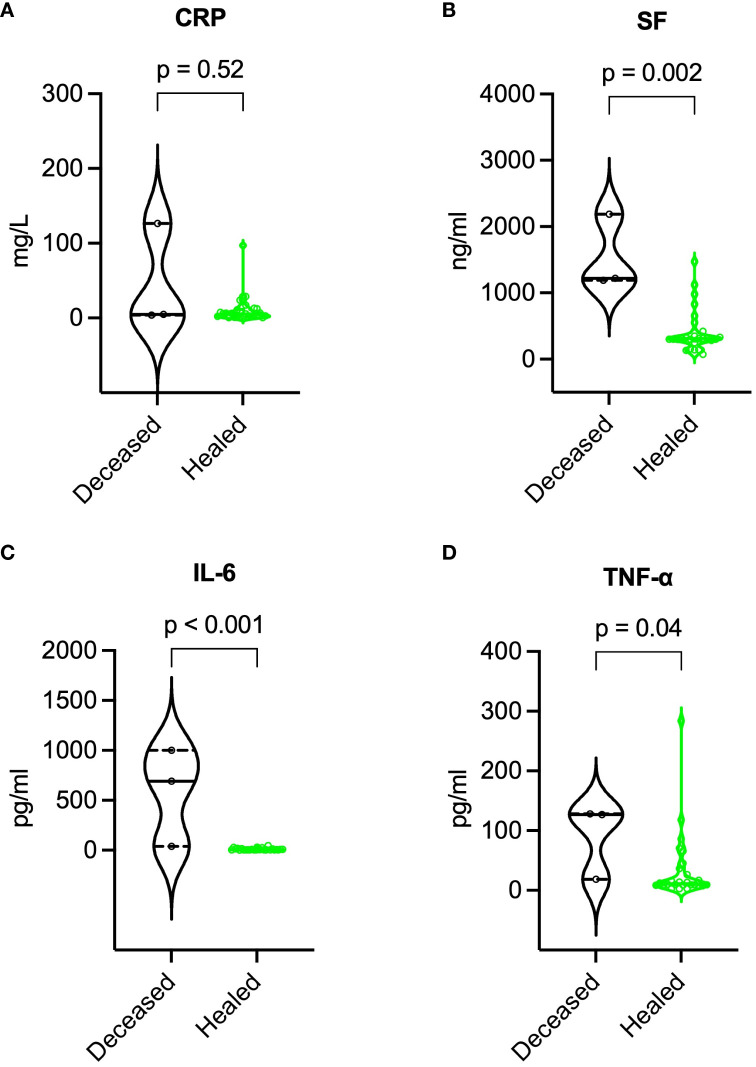
Comparison of inflammatory markers during recovery between deceased and healed patients. **(A)** CRP, **(B)** SF, **(C)** IL-6, and **(D)** TNF-α. P values were determined by Mann-Whitney U test. CRP, C-reactive protein; IL-6, interleukin-6; SF, serum ferritin; TNF-α, tumor necrosis factor-α.

**Figure 8:**
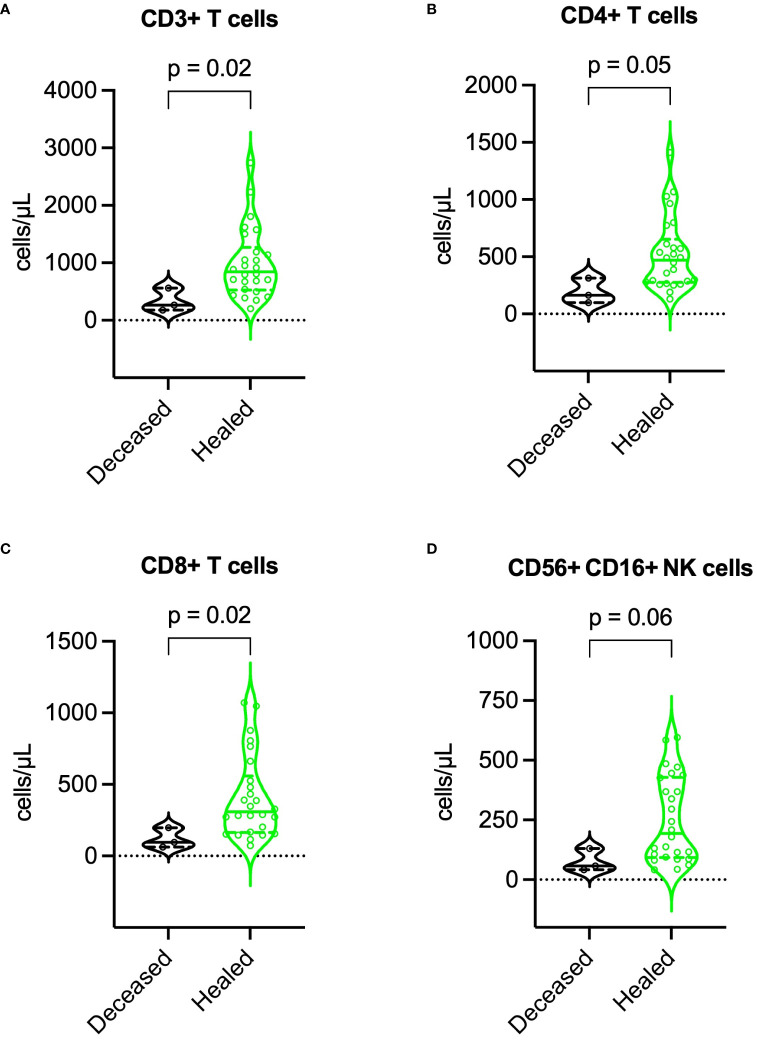
Comparison of lymphocyte subset counts during recovery between deceased and healed patients. **(A)** CD3^+^ T cells, **(B)** CD4^+^ T cells, **(C)** CD8^+^ T cells, and **(D)** NK cells. P values were determined by Mann-Whitney U test. NK, natural killer.

Compared to survivors, deceased patients exhibited lower BMI (16.8 vs. 23.8 kg/m², p = 0.003), higher SCORTEN (5 vs. 3, p = 0.041), more extensive epidermal detachment (40% vs. 8% of BSA, p = 0.033), higher baseline SF, IL-6, and TNF-α levels, lower lymphocyte subset counts (especially NK cells), and higher incidence of major adverse events. For further details, refer to **Table 3**. During recovery, deceased patients maintained higher SF, IL-6, and TNF-α levels, and lower lymphocyte subset counts. More information is available in **Figures 7**, **8**.

**Table 3:** Comparison of baseline characteristics and major treatment-related adverse events between deceased and healed patients with immune-related epidermal necrolysis.

	Deceased (n = 3)	Healed (n = 29)	p value
Age, years	55 ± 4	59 ± 12	0.542
Sex, male	1 (33%)	17 (59%)	0.568
Body mass index, kg/m^2^	16.8 ± 4.4	23.8 ± 3.4	0.003
Malignancies			
Lung cancer	1 (33%)	12 (41%)	0.605
Gastrointestinal cancer	1 (33%)	8 (28%)	
Hepatocellular carcinoma	0	6 (21%)	
Other malignancies	1 (33%)	3 (10%)	
Culprit ICIs			
Sintilimab	2 (67%)	11 (39%)	0.574
Pembrolizumab	1 (33%)	6 (21%)	
Camrelizumab	0	5 (17%)	
Other ICIs	0	7 (24%)	
Cycles from ICI start to onset	1 (1, 2)	1 (1, 3)	0.669
Latency from ICI start to onset, days	14 (13, 23)	17 (8, 57)	0.855
Concurrent drugs or therapy within 4 weeks			
Chemotherapy	3 (100%)	14 (48%)	0.229
Other treatments	0	15 (52%)	
Clinical manifestations			
Stevens-Johnson syndrome	0	21 (72%)	0.033
Toxic epidermal necrolysis	3 (100%)	8 (28%)	
Patients with fever	2 (67%)	24 (83%)	0.476
Patients with mucositis	2 (67%)	25 (86%)	0.410
Epidermal detachment, % of BSA	40 (38, 45)	8 (4, 28)	0.033
SCORTEN (range 0-7)	5 (4, 6)	3 (2, 3)	0.041
Laboratory findings			
C-reactive protein, mg/L	38.6 (30.5, 116.6)	34.2 (20.0, 54.0)	0.580
Serum ferritin, ng/ml	844 (811, 1264)	780 (435, 933)	0.258
Interleukin-6, pg/ml	37.4 (31.8, 236.2)	21.1 (7.7, 45.2)	0.146
Tumor necrosis factor-α, pg/ml	31.0 (23.6, 63.6)	18.5 (13.3, 29.8)	0.343
CD3^+^ T cell count, /µL	279 (235, 499)	449 (333, 732)	0.383
CD4^+^ T cell count, /µL	159 (132, 311)	225 (168, 433)	0.383
CD8^+^ T cell count, /µL	73 (69, 136)	162 (110, 255)	0.185
Natural killer cell count, /µL	46 (43, 56)	111 (73, 211)	0.033
Treatments and major adverse events			
Prednisone equivalent dose, mg/kg/day	2.4 (2.1, 2.5)	1.4 (1.3, 1.7)	0.004
IVIG cumulative dose, g/kg	6.8 (5.3, 7.9)	3.0 (2.8, 3.6)	0.024
Infections	2 (67%)	5 (17%)	0.113
Disseminated intravascular coagulation	3 (100%)	0	< 0.001
Gastrointestinal bleeding	2 (67%)	0	0.006
Respiratory failure	2 (67%)	0	0.006

Data are presented as mean ± standard deviation, median (25th and 75th percentiles), or counts (percentages) as appropriate. P values were calculated using two-tailed t-test for normally distributed continuous variables, Mann-Whitney U test for non-normally distributed continuous variables, and Fisher's exact test or Fisher-Freeman-Halton exact test for categorical variables. Abbreviations: BSA, body surface area; ICIs, immune checkpoint inhibitors; IVIG, intravenous immunoglobulin; SCORTEN, severity of illness score for toxic epidermal necrolysis.

## 4 Discussion

Our research offers new perspectives on the management of irEN. Primarily, it reveals that adjunctive use of TNFi with corticosteroids significantly accelerates re-epithelization, mitigates corticosteroid exposure, and reduces acute mortality risk without increasing major adverse events. We also demonstrated that re-epithelization time was negatively correlated with BMI, and positively correlated with the extent of epidermal detachment, and levels of acute-phase IL-6 and TNF-α. Additionally, the dynamics of inflammatory markers and lymphocyte subsets highlight their value as biomarkers for monitoring disease activity and tailoring treatment regimens. Finally, our research has identified several prognostic indicators for mortality, such as persistent elevation of inflammatory markers, ongoing reduction in lymphocyte subsets, and the presence of serious complications like disseminated intravascular coagulation and respiratory failure.

Our study found that irEN develops early during ICI therapy—typically after just one cycle and with a median latency of 16 days, showing no notable gender disparity in incidence. The clinical manifestations of irEN, characterized by extensive erythema and epidermal detachment, accompanied by high fever and severe mucositis, are consistent with epidermal necrolysis caused by conventional medications (18–21). Following intensive management, most patients recovered within 2-3 weeks, while a minority succumbed within 30 days. These observations highlight the critical nature of epidermal necrolysis as a potentially fatal irAE, necessitating vigilant monitoring from the initial stages of ICI therapy.

Our study represents the first prospective cohort study evaluating the effectiveness and safety of combining corticosteroids with TNFi in the treatment of irEN. Our findings suggest that, this synergistic approach significantly accelerates re-epithelization, reduces corticosteroid exposure, and decreases the mortality risks, without increasing the adverse events like infections or gastrointestinal bleeding. TNF-α may play an essential role in the pathogenesis of epidermal necrolysis, where activated CD8^+^ T cells induce widespread keratinocyte death, resulting in the substantial release of pro-inflammatory cytokines, such as TNF-α, which in turn amplifies both local and systemic inflammatory responses (22). Elevated levels of TNF-α have been detected in serum and blister fluid, along with overexpression in the lesional skin of patients with epidermal necrolysis (23–27). The administration of etanercept, a TNFi, has been shown to markedly reduce plasma TNF-α levels and diminish epidermal matrix metalloproteinase 9 expression, potentially disrupting a key pathological pathway in the development of epidermal necrolysis (16, 28, 29). Supporting this, the work of Wang et al. in a randomized controlled trial revealed that etanercept reduces re-epithelization time versus corticosteroid monotherapy (14 vs. 17 days), particularly in those with TEN (14 vs. 19 days) (16). Furthermore, Zhang et al.'s multicenter retrospective study indicated superior outcomes with the combination of corticosteroids and etanercept in terms of re-epithelization time, acute mortality, and incidence of gastrointestinal bleeding when compared to corticosteroids alone (30). Similarly, Tian et al. reported that combining etanercept with corticosteroids and IVIG significantly decreased the length of hospital stay (14 vs. 19 days) and corticosteroid dosage (925 vs. 1413 mg) (31). Systematic reviews and meta-analyses further suggest that etanercept may reduce mortality rates in patients with epidermal necrolysis when compared to corticosteroids (32, 33). Our study is the first to provide preliminary evidence for the effectiveness and safety of combining TNFi with corticosteroids in treating irEN.

The safety of TNFi in the treatment of irAEs remains an active field of investigation. Current evidence suggests that the use of TNFi in patients with malignancies may be safe. Preclinical studies and case reports indicate that TNFi might not only ameliorate irAEs but also enhance the antitumor effects of ICIs (10–13). On the other hand, in patients with inflammatory diseases and a history of malignancy, the cancer recurrence rate was similar between those treated with TNFi and those who were not on immunosuppressive therapy (34). Furthermore, meta-analyses have reported that the administration of TNFi at recommended dosages does not increase the risk of infections or death in patients with inflammatory disorders (35, 36). In our study, the incidence of major adverse events did not differ significantly between groups. However, infections occurred earlier in the combination group than in the corticosteroid group. These results imply that application of TNFi for irEN might be safe, though vigilant monitoring for infections remains imperative.

Research into the factors that affect the duration of re-epithelization in epidermal necrolysis has been scant. Ao et al. found a positive correlation between re-epithelization time and the serum levels of IL-6 and IL-15 (37). Our research adds new insights by uncovering a negative correlation between re-epithelization time and BMI, and a positive correlation with both the epidermal detachment area and the serum levels of IL-6 and TNF-α. These observations suggest that interventions focused on enhancing nutritional status, mitigating inflammatory responses, and preventing extensive epidermal necrosis could be pivotal in accelerating the re-epithelization process, thereby potentially improving patient outcomes.

Our study also highlights SF, IL-6, TNF-α, and lymphocyte subsets as potential biomarkers of disease activity in irEN patients. During the acute phase, we observe an increase in these inflammatory markers coupled with a decline in lymphocyte subsets. This trend reverses during recovery, with a significant drop in inflammatory markers and a rebound in lymphocyte subset counts. These dynamics offer clinicians valuable indicators for assessing disease activity and tailoring treatment regimens more precisely. Considering the aggressive nature of epidermal necrolysis, where peak detachment typically occurs within a median of 8 days (38), prompt administration of high-dose corticosteroids is crucial. Supplementing this initial management with TNFi can halt the inflammatory cascade, limit epidermal necrosis, and accelerate recovery. Nonetheless, a prolonged course of corticosteroids could impair wound healing and elevate infection risks (39). Thus, it is recommended to initiate corticosteroid tapering as signs of disease stabilization appear, characterized by the absence of new erythema and blistering, no further epidermal detachment, along with a concurrent decrease in inflammatory markers and a restoration of lymphocyte subset counts.

This study has pinpointed potential risk factors associated with mortality in patients with irEN, including low BMI, high SCORTEN, sustained elevation of inflammatory markers, ongoing reduction in lymphocyte subset counts, and severe complications such as disseminated intravascular coagulation, gastrointestinal bleeding, or respiratory failure. Insight from a retrospective analysis of over 50,000 cases of epidermal necrolysis revealed that older age, chronic kidney disease, pneumonia, sepsis, and malignancies stand as independent predictors of mortality (40). Notably, disseminated intravascular coagulation was significantly linked to systemic complications including gastrointestinal bleeding, multiorgan failure, and infection, culminating in a substantial escalation of mortality risk (78% vs. 7%) (41). Furthermore, in patients with TEN who required mechanical ventilation due to respiratory failure, the mortality rate increased to 57% (42). Our findings also imply that factors such as low BMI, consistent rise in inflammatory markers, and a persistent decline in lymphocyte subset counts may contribute to a higher risk of mortality.

Our study has several limitations. Primarily, as a single-center observational study, it lacks the robustness of randomization and blinding. The relatively small sample size also hindered us to perform regression analysis for identifying independent mortality predictors. Additionally, the concurrent administration of IVIG in patients with TEN might have influenced the re-epithelization time and other outcomes. Despite this, there were no significant differences in either the proportion of patients receiving IVIG or the total doses administered between the combination and corticosteroid groups, preserving the observed trend of a faster re-epithelization in the combination group. Moreover, the complexity of determining causality between ICIs and epidermal necrolysis is heightened by the patients’ concurrent medications prior to onset, including antibiotics and targeted agents. Antibiotics are widely recognized as a class of high-risk medications to induce epidermal necrolysis (43). Furthermore, recent literature has started to identify targeted agents, such as osimertinib and sorafenib (44–46), as potential culprits in this serious condition. In instances where epidermal necrolysis develops alongside the administration of ICIs and these drugs, it is possible that both agents collectively contribute to the pathogenesis (47). Finally, our use of SCORTEN as a benchmark to evaluate treatment impact on acute mortality has its limitations. Notably, SCORTEN may overestimate mortality at higher score ranges, potentially resulting in a positively biased estimate of the efficacy of any intervention (48).

## 5 Conclusion

Our study has provided valuable insights into the management of irEN, a potentially life-threatening condition following immunotherapy. The findings demonstrate that combining corticosteroids with TNFi significantly shortens re-epithelization time, reduces corticosteroid exposure, and lowers acute mortality rates without increasing major treatment-related adverse events. We also identified potential biomarkers indicative of disease activity and prognosis, especially inflammatory markers and lymphocyte subsets, offering clinicians useful tools for monitoring and adjusting treatment regimens. Given the study's inherent limitations, including its single-center, observational design with a small sample size, we advocate for larger-scale, multi-center, randomized controlled trials to validate our findings. Such comprehensive research is crucial to optimize treatment protocols and improve patient care in the era of cancer immunotherapy.

## Data availability statement

The original contributions presented in the study are included in the article/**Supplementary Material**. Further inquiries can be directed to the corresponding author.

## Ethics statement

The studies involving humans were approved by Ethics Committee of Peking Union Medical College Hospital. The studies were conducted in accordance with the local legislation and institutional requirements. The participants provided their written informed consent to participate in this study. Written informed consent was obtained from the individual(s) for the publication of any potentially identifiable images or data included in this article.

## Author contributions

C-XH: Conceptualization, Data curation, Formal analysis, Funding acquisition, Investigation, Methodology, Project administration, Validation, Visualization, Writing – original draft, Writing – review & editing, Resources. LG: Investigation, Writing – original draft, Writing – review & editing, Resources, Data curation, Formal analysis, Validation, Visualization. TQ: Investigation, Supervision, Writing – original draft, Writing – review & editing, Conceptualization, Resources. H-ZJ: Writing – original draft, Writing – review & editing, Conceptualization, Funding acquisition, Project administration, Supervision.
