# Supplementary material for: Tumor necrosis factor inhibitors enhance corticosteroid therapy for Stevens-Johnson syndrome and toxic epidermal necrolysis linked to immune checkpoint inhibitors: a prospective study
Source: Front Immunol. 2024 Aug 7;15:1421684. doi: 10.3389/fimmu.2024.1421684 (PMC11335491; doi:10.3389/fimmu.2024.1421684)
Supplement: Supplementary file 1 [file Table_1.docx]

Supplementary Tables

Supplementary Table 1 Baseline demographics and cancer treatment details in patients with immune-related epidermal necrolysis.

| **Patient** | **Group** | **Diagnosis** | **Sex** | **Age, y** | **BMI** | **Malignancies** | **ICIs** | **Cycles** | **Latency, d** | **Concurrent therapies** |
| --- | --- | --- | --- | --- | --- | --- | --- | --- | --- | --- |
| 1 | Combination | TEN | Male | 51 | 12.8 | Esophageal cancer | Pembrolizumab | 2 | 32 | Docetaxel, carboplatin |
| 2 | Combination | TEN | Female | 59 | 21.5 | Lung cancer | Sintilimab | 1 | 12 | Gemcitabine |
| 3 | Combination | TEN | Male | 63 | 26.1 | Lung cancer | Sintilimab | 1 | 5 | Radiotherapy |
| 4 | Combination | TEN | Male | 60 | 21.0 | Gastric cancer | Sintilimab | 1 | 8 | Biapenem |
| 5 | Combination | TEN | Female | 56 | 25.4 | Lung cancer | Camrelizumab | 3 | 50 | Anlotinib |
| 6 | Combination | SJS | Male | 63 | 22.0 | Retroperitoneal carcinoma | Sintilimab | 2 | 53 | Paclitaxel, carboplatin |
| 7 | Combination | SJS | Female | 58 | 24.8 | Lung cancer | Serplulimab | 3 | 54 | Carboplatin, etoposide |
| 8 | Combination | SJS | Female | 66 | 23.4 | Hepatocellular carcinoma | Toripalimab | 1 | 7 | Oxaliplatin, gemcitabine |
| 9 | Combination | SJS | Male | 57 | 26.9 | Scrotum adenocarcinoma | Pembrolizumab | 2 | 52 | Bevacizumab, paclitaxel, carboplatin |
| 10 | Combination | SJS | Male | 64 | 24.6 | Gastrointestinal cancer | Pembrolizumab | 1 | 11 | Docetaxel, carboplatin |
| 11 | Combination | SJS | Male | 58 | 24.2 | Lung cancer | Pembrolizumab | 1 | 14 | Bevacizumab, pemetrexed, carboplatin |
| 12 | Combination | SJS | Male | 70 | 20.5 | Lung cancer | Sintilimab | 2 | 68 | Paclitaxel, carboplatin |
| 13 | Combination | SJS | Female | 68 | 23.4 | Gastrointestinal cancer | Camrelizumab | 1 | 11 | Fruquintinib |
| 14 | Combination | SJS | Female | 74 | 25.6 | Lung cancer | Pembrolizumab | 1 | 6 | Paclitaxel, carboplatin |
| 15 | Corticosteroid | TEN | Female | 55 | 16.0 | Thymoma | Sintilimab | 1 | 14 | Doxorubicin |
| 16 | Corticosteroid | TEN | Male | 70 | 17.8 | Lung cancer | Sintilimab | 2 | 37 | Paclitaxel, cisplatin |
| 17 | Corticosteroid | TEN | Male | 73 | 22.4 | Lung cancer | Sintilimab | 1 | 20 | Paclitaxel, carboplatin |
| 18 | Corticosteroid | TEN | Male | 71 | 20.7 | Esophageal cancer | Camrelizumab | 1 | 7 | Cefoperazone/sulbactam |
| 19 | Corticosteroid | TEN | Male | 52 | 22.0 | Esophageal cancer | Sintilimab | 1 | 60 | Radiotherapy |
| 20 | Corticosteroid | TEN | Female | 73 | 16.7 | Esophageal cancer | Durvalumab | 1 | 15 | Docetaxel, carboplatin |
| 21 | Corticosteroid | SJS | Male | 57 | 17.3 | Gastrointestinal cancer | Sintilimab | 5 | 155 | Bevacizumab |
| 22 | Corticosteroid | SJS | Female | 34 | 25.3 | Lung cancer | Pembrolizumab | 1 | 3 | Paclitaxel, carboplatin |
| 23 | Corticosteroid | SJS | Female | 59 | 26.6 | Lung cancer | Sintilimab | 16 | 530 | Chinese herb |
| 24 | Corticosteroid | SJS | Female | 67 | 24.8 | Hepatocellular carcinoma | Camrelizumab | 1 | 6 | Sorafenib |
| 25 | Corticosteroid | SJS | Female | 57 | 25.7 | Hepatocellular carcinoma | Tislelizumab | 1 | 10 | Sorafenib |
| 26 | Corticosteroid | SJS | Male | 48 | 21.6 | Malignant mesothelioma | Ipilimumab | 3 | 148 | Nivolumab |
| 27 | Corticosteroid | SJS | Female | 64 | 24.0 | Lung cancer | Pembrolizumab | 3 | 95 | Paclitaxel, carboplatin |
| 28 | Corticosteroid | SJS | Male | 67 | 33.0 | Hepatocellular carcinoma | Tislelizumab | 12 | 242 | Donafenib |
| 29 | Corticosteroid | SJS | Male | 45 | 24.6 | Hepatocellular carcinoma | Camrelizumab | 3 | 45 | Regorafenib |
| 30 | Corticosteroid | SJS | Male | 60 | 24.2 | Lung cancer | Sintilimab | 1 | 17 | Paclitaxel, cisplatin |
| 31 | Corticosteroid | SJS | Male | 29 | 24.9 | Hepatocellular carcinoma | Tislelizumab | 1 | 4 | Donafenib |
| 32 | Corticosteroid | SJS | Female | 35 | 29.7 | Gastrointestinal cancer | Sintilimab | 1 | 9 | Regorafenib |

Abbreviations: BMI, body mass index; ICIs, immune checkpoint inhibitors; SJS, Stevens-Johnson syndrome; TEN, toxic epidermal necrolysis.

**Supplementary Table 2** Clinical manifestations and treatment regimens of the patients with immune-related epidermal necrolysis.

| **Patient** | **Group** | **Tmax, ℃** | **Mucositis** | **Epidermal detachment, % of BSA** | **SCORTEN** | **Prednisone equivalent dose, mg/kg/d** | **Prednisone cumulative dose, mg** | **Corticosteroid duration, d** | **TNFi** | **IVIG dose, g/kg** |
| --- | --- | --- | --- | --- | --- | --- | --- | --- | --- | --- |
| 1 | Combination | 40.0 | Yes | 50 | 5 | 2.4 | 1225 | 16 | Infliximab | 9.0 |
| 2 | Combination | NA | No | 40 | 6 | 1.8 | 1600 | 19 | Infliximab | 3.8 |
| 3 | Combination | 39.5 | Yes | 90 | 4 | 1.9 | 2579 | 35 | Infliximab | 2.8 |
| 4 | Combination | 38.8 | Yes | 35 | 4 | 1.8 | 1210 | 18 | Infliximab | 3.0 |
| 5 | Combination | 39.1 | Yes | 30 | 5 | 1.5 | 1165 | 20 | Infliximab | 3.1 |
| 6 | Combination | 38.8 | No | 8 | 3 | 1.7 | 1280 | 26 | Etanercept | NA |
| 7 | Combination | NA | Yes | 9 | 2 | 1.4 | 1125 | 20 | Etanercept | NA |
| 8 | Combination | 39.2 | Yes | 8 | 3 | 1.4 | 825 | 19 | Etanercept | NA |
| 9 | Combination | 38.6 | Yes | 8 | 3 | 1.3 | 760 | 14 | Etanercept | NA |
| 10 | Combination | 39.5 | Yes | 10 | 3 | 1.4 | 1490 | 26 | Etanercept | NA |
| 11 | Combination | 38.8 | Yes | 3 | 3 | 1.1 | 825 | 20 | Etanercept | NA |
| 12 | Combination | 37.7 | Yes | 5 | 2 | 1.5 | 1130 | 22 | Etanercept | NA |
| 13 | Combination | 39.0 | Yes | 1 | 4 | 1.0 | 530 | 22 | Etanercept | NA |
| 14 | Combination | 37.5 | Yes | 1 | 3 | 1.5 | 1200 | 25 | Etanercept | NA |
| 15 | Corticosteroid | 38.8 | Yes | 35 | 3 | 2.7 | 1815 | 27 | NA | 6.8 |
| 16 | Corticosteroid | NA | Yes | 40 | 3 | 1.8 | 3190 | 58 | NA | 5.5 |
| 17 | Corticosteroid | 40.0 | Yes | 50 | 4 | 2.0 | 1990 | 25 | NA | 2.9 |
| 18 | Corticosteroid | 38.6 | Yes | 80 | 3 | 1.8 | 2125 | 38 | NA | 3.8 |
| 19 | Corticosteroid | 39.0 | Yes | 25 | 4 | 1.6 | 1058 | 22 | NA | 1.9 |
| 20 | Corticosteroid | NA | No | 35 | 3 | 1.3 | 2180 | 59 | NA | 3.0 |
| 21 | Corticosteroid | 39.0 | Yes | 5 | 2 | 2.0 | 1220 | 34 | NA | NA |
| 22 | Corticosteroid | 39.1 | Yes | 1 | 2 | 1.3 | 765 | 24 | NA | NA |
| 23 | Corticosteroid | NA | Yes | 5 | 2 | 1.3 | 1580 | 26 | NA | NA |
| 24 | Corticosteroid | 39.0 | Yes | 5 | 2 | 1.5 | 1595 | 33 | NA | NA |
| 25 | Corticosteroid | 39.8 | Yes | 1 | 2 | 1.4 | 1005 | 21 | NA | NA |
| 26 | Corticosteroid | NA | Yes | 5 | 2 | 1.6 | 1525 | 39 | NA | NA |
| 27 | Corticosteroid | 37.8 | Yes | 10 | 3 | 1.7 | 930 | 22 | NA | NA |
| 28 | Corticosteroid | 39.6 | Yes | 5 | 3 | 1.1 | 1430 | 24 | NA | NA |
| 29 | Corticosteroid | 39.8 | No | 1 | 2 | 1.4 | 1160 | 24 | NA | NA |
| 30 | Corticosteroid | 39.0 | No | 8 | 2 | 1.4 | 2395 | 46 | NA | NA |
| 31 | Corticosteroid | 38.5 | Yes | 5 | 1 | 1.2 | 1005 | 24 | NA | NA |
| 32 | Corticosteroid | 39.5 | Yes | 2 | 1 | 1.3 | 1940 | 31 | NA | NA |

Abbreviations: BSA, body surface area; IVIG, intravenous immunoglobulin; NA, not applicable; SCORTEN, severity-of-illness score for toxic epidermal necrolysis; Tmax, maximum body temperature; TNFi, tumor necrosis factor inhibitors.

**Supplementary Table 3** Treatment-related adverse events and outcomes of the patients with immune-related epidermal necrolysis.

| **Patient** | **Group** | **Outcome** | **Re-epithelization time, d‎** | **Infections** | **Infection time, d** | **DIC** | **Gastrointestinal bleeding** | **Respiratory failure** | **Death time, d** | **Cause of death** |
| --- | --- | --- | --- | --- | --- | --- | --- | --- | --- | --- |
| 1 | Combination | Deceased | NA | Pneumonia | 5 | Yes | Yes | Yes | 15 | Septic shock |
| 2 | Combination | Deceased | NA | NA | NA | Yes | No | No | 20 | DIC |
| 3 | Combination | Healed | 19 | Sepsis | 8 | No | No | No | NA |  |
| 4 | Combination | Healed | 20 | NA | NA | No | No | No | NA |  |
| 5 | Combination | Healed | 14 | NA | NA | No | No | No | NA |  |
| 6 | Combination | Healed | 14 | Pneumonia | 20 | No | No | No | NA |  |
| 7 | Combination | Healed | 16 | NA | NA | No | No | No | NA |  |
| 8 | Combination | Healed | 12 | NA | NA | No | No | No | NA |  |
| 9 | Combination | Healed | 12 | NA | NA | No | No | No | NA |  |
| 10 | Combination | Healed | 15 | NA | NA | No | No | No | NA |  |
| 11 | Combination | Healed | 17 | NA | NA | No | No | No | NA |  |
| 12 | Combination | Healed | 14 | NA | NA | No | No | No | NA |  |
| 13 | Combination | Healed | 12 | NA | NA | No | No | No | NA |  |
| 14 | Combination | Healed | 12 | NA | NA | No | No | No | NA |  |
| 15 | Corticosteroid | Deceased | NA | Pneumonia | 20 | Yes | Yes | Yes | 26 | Septic shock |
| 16 | Corticosteroid | Healed | 39 | Pneumonia | 12 | No | No | No | NA |  |
| 17 | Corticosteroid | Healed | 37 | Sepsis | 21 | No | No | No | NA |  |
| 18 | Corticosteroid | Healed | 43 | NA | NA | No | No | No | NA |  |
| 19 | Corticosteroid | Healed | 22 | NA | NA | No | No | No | NA |  |
| 20 | Corticosteroid | Healed | 44 | NA | NA | No | No | No | NA |  |
| 21 | Corticosteroid | Healed | 24 | Pneumonia | 32 | No | No | No | NA |  |
| 22 | Corticosteroid | Healed | 15 | NA | NA | No | No | No | NA |  |
| 23 | Corticosteroid | Healed | 18 | NA | NA | No | No | No | NA |  |
| 24 | Corticosteroid | Healed | 20 | NA | NA | No | No | No | NA |  |
| 25 | Corticosteroid | Healed | 14 | NA | NA | No | No | No | NA |  |
| 26 | Corticosteroid | Healed | 18 | NA | NA | No | No | No | NA |  |
| 27 | Corticosteroid | Healed | 21 | NA | NA | No | No | No | NA |  |
| 28 | Corticosteroid | Healed | 18 | NA | NA | No | No | No | NA |  |
| 29 | Corticosteroid | Healed | 14 | NA | NA | No | No | No | NA |  |
| 30 | Corticosteroid | Healed | 22 | NA | NA | No | No | No | NA |  |
| 31 | Corticosteroid | Healed | 21 | NA | NA | No | No | No | NA |  |
| 32 | Corticosteroid | Healed | 14 | NA | NA | No | No | No | NA |  |

Abbreviations: DIC, disseminated intravascular coagulation; NA, not applicable.

**Supplementary Table 4** Comparison of baseline laboratory findings between groups in patients with immune-related epidermal necrolysis.

|  | **Total cohort**  **(n = 32)** | **Combination group**  **(n = 14)** | **Corticosteroid group**  **(n = 18)** | **Normal range** | **p value** |
| --- | --- | --- | --- | --- | --- |
| **Complete blood cell count** | | | | | |
| WBC, × 10^9^/L | 5.03 ± 2.77 | 4.78 ± 2.99 | 5.22 ± 2.66 | 3.5 ~ 9.5 | 0.665 |
| Neutrophils, × 10^9^/L | 3.69 ± 2.35 | 3.61 ± 2.83 | 3.75 ± 1.98 | 2.0 ~ 7.5 | 0.871 |
| Lymphocytes, × 10^9^/L | 0.77 ± 0.46 | 0.69 ± 0.47 | 0.85 ± 0.45 | 0.8 ~ 4.0 | 0.332 |
| Hemoglobin, g/L | 115 ± 21 | 107 ± 22 | 122 ± 19 | 120 ~ 160 | 0.043 |
| Platelets, × 10^9^/L | 191 ± 106 | 194 ± 115 | 189 ± 102 | 100 ~ 350 | 0.900 |
| **Blood biochemistry** | | | | | |
| ALT, U/L | 28 (14, 40) | 26 (16, 34) | 32 (12, 48) | 9 ~ 50 | 0.955 |
| Total bilirubin, μmol/L | 9.0 (7.4, 15.4) | 9.6 (7.0, 13.3) | 8.5 (7.4, 32.6) | 5.1 ~ 22.2 | 0.896 |
| Albumin, g/L | 37 ± 5 | 36 ± 6 | 39 ± 4 | 35 ~ 52 | 0.119 |
| Creatinine, μmol/L | 66 ± 23 | 75 ± 23 | 59 ± 20 | 59 ~ 104 | 0.054 |
| Glucose, mmol/L | 8.6 ± 4.0 | 8.9 ± 3.4 | 8.3 ± 4.6 | 3.9 ~ 6.1 | 0.690 |
| **Inflammatory markers** | | | | | |
| CRP, mg/L | 34.3 (22.2, 54.0) | 32.7 (20.8, 90.0) | 38.5 (21.1, 53.9) | < 3.0 | 0.896 |
| SF, ng/ml | 785 (448, 935) | 689 (433, 865) | 825 (452, 1038) | 24 ~ 336 | 0.338 |
| IL-6, pg/ml | 25.0 (8.0, 45.3) | 12.2 (7.8, 50.9) | 27.8 (8.0, 51.1) | < 5.9 | 0.633 |
| TNF-α, pg/ml | 18.7 (13.6, 33.0) | 21.0 (11.2, 33.9) | 18.4 (15.5, 28.0) | < 8.1 | 0.772 |
| **Peripheral blood lymphocyte subsets** | | | | | |
| CD3^+^ T cells, /µL | 433 (314, 707) | 380 (209, 520) | 499 (349, 818) | 940 ~ 2140 | 0.135 |
| CD4^+^ T cells, /µL | 212 (161, 436) | 201 (135, 273) | 274 (177, 536) | 550 ~ 1200 | 0.135 |
| CD8^+^ T cells, /µL | 161 (104, 243) | 126 (71, 227) | 190 (110, 259) | 380 ~ 790 | 0.220 |
| CD56^+^ CD16^+^ NK cells, /µL | 105 (68, 205) | 156 (59, 239) | 97 (70, 154) | 155 ~ 550 | 0.482 |

Data are presented as mean ± standard deviation and median (25th and 75th percentiles) as appropriate. P values were calculated using two-tailed t-test for normally distributed continuous variables and Mann-Whitney U test for non-normally distributed continuous variables. Abbreviations: ALT, alanine aminotransferase; CRP, C-reactive protein; IL-6, interleukin-6; NK, natural killer; SF, serum ferritin; TNF-α, tumor necrosis factor-α; WBC, white blood cell.

**Supplementary Table 5** Comparison of baseline characteristics and outcomes between the patients with immune-related Stevens-Johnson syndrome and toxic epidermal necrolysis.

|  | **SJS (n = 21)** | **TEN (n = 11)** | **p value** |
| --- | --- | --- | --- |
| Age, years | 57 ± 12 | 62 ± 8 | 0.243 |
| Sex, male | 11 (52%) | 7 (64%) | 0.712 |
| Body mass index, kg/m^2^ | 24.6 ± 3.1 | 20.2 ± 4.0 | 0.002 |
| **Malignancies** |  |  |  |
| Lung cancer | 8 (38%) | 5 (46%) | 0.146 |
| Gastrointestinal cancer | 4 (19%) | 5 (46%) |  |
| Hepatocellular carcinoma | 6 (29%) | 0 |  |
| Others | 3 (14%) | 1 (9%) |  |
| **Culprit ICIs** |  |  |  |
| Pembrolizumab | 6 (29%) | 1 (9%) | 0.223 |
| Camrelizumab | 3 (14%) | 2 (18%) |  |
| Sintilimab | 6 (29%) | 7 (64%) |  |
| Others | 6 (29%) | 1 (9%) |  |
| Cycles from ICI start to onset | 1 (1, 3) | 1 (1, 2) | 0.750 |
| Latency from ICI start to onset, days | 17 (8, 82) | 15 (8, 37) | 0.488 |
| **Concurrent treatments** | | | |
| Chemotherapy | 11 (52%) | 6 (55%) | 1.000 |
| Others | 10 (48%) | 5 (46%) |  |
| **Clinical manifestations** |  |  |  |
| Patients with fever | 18 (86%) | 8 (73%) | 0.390 |
| Peak temperature, ℃ | 38.9 ± 0.7 | 39.2 ± 0.5 | 0.247 |
| Patients with mucositis | 18 (86%) | 9 (82%) | 1.000 |
| Epidermal detachment, % of BSA | 5 (2, 8) | 40 (35, 50) | < 0.001 |
| SCORTEN (range 0-7) | 2 (2, 3) | 4 (3, 5) | < 0.001 |
| **Laboratory findings** |  |  |  |
| WBC, ×10^9^/L | 5.70 ± 2.70 | 3.75 ± 2.54 | 0.057 |
| Lymphocytes, ×10^9^/L | 0.93 ± 0.47 | 0.47 ± 0.23 | 0.005 |
| Hemoglobin, g/L | 123 ± 21 | 100 ± 13 | 0.002 |
| Albumin, g/L | 39 ± 4 | 33 ± 6 | < 0.001 |
| CRP, mg/L | 34.2 (22.6, 47.6) | 38.6 (18.0, 97.5) | 0.457 |
| SF, ng/ml | 665 (413, 830) | 1014 (712, 1298) | 0.031 |
| IL-6, pg/ml | 14.0 (8.1, 37.0) | 37.4 (7.6, 86.1) | 0.158 |
| TNF-α, pg/ml | 17.5 (12.0, 20.5) | 34.4 (25.4, 36.8) | 0.003 |
| CD3^+^ T cells, /µL | 489 (379, 948) | 324 (190, 509) | 0.038 |
| CD4^+^ T cells, /µL | 242 (175, 627) | 183 (124, 322) | 0.074 |
| CD8^+^ T cells, /µL | 205 (119, 276) | 109 (65, 199) | 0.046 |
| CD56^+^ CD16^+^ NK cells, /µL | 155 (90, 251) | 66 (39, 100) | 0.002 |
| **Treatment regimens and outcomes** |  |  |  |
| Prednisone equivalent dose, mg/kg/d | 1.4 (1.3, 1.5) | 1.8 (1.6, 2.0) | < 0.001 |
| Re-epithelization time, days | 15 (14, 19) | 30 (19, 42) | 0.003 |
| Observed mortality rate, % | 0 | 27 | 0.033 |

Data are presented as mean ± standard deviation, median (25th and 75th percentiles), or counts (percentages) as appropriate. P values were calculated using two-tailed t-test for normally distributed continuous variables, Mann-Whitney U test for non-normally distributed continuous variables, and Fisher's exact test or Fisher-Freeman-Halton exact test for categorical variables. Abbreviations: BSA, body surface area; CRP, C-reactive protein; ICIs, immune checkpoint inhibitors; IL-6, interleukin-6; NK, natural killer; SCORTEN, severity-of-illness score for toxic epidermal necrolysis; SF, serum ferritin; SJS, Stevens-Johnson syndrome; TEN, toxic epidermal necrolysis; TNF-α, tumor necrosis factor-α; WBC, white blood cell.
